# Evaluating the Effectiveness of 2D and 3D CT Image Features for Predicting Tumor Response to Chemotherapy

**DOI:** 10.3390/bioengineering10111334

**Published:** 2023-11-20

**Authors:** Neman Abdoli, Ke Zhang, Patrik Gilley, Xuxin Chen, Youkabed Sadri, Theresa Thai, Lauren Dockery, Kathleen Moore, Robert Mannel, Yuchen Qiu

**Affiliations:** 1 School of Electrical and Computer Engineering, University of Oklahoma, Norman, OK 73019, USA; neman.abdoli@ou.edu (N.A.); kezhang@ou.edu (K.Z.); youkabed.sadri@ou.edu (Y.S.); 2 Stephenson School of Biomedical Engineering, University of Oklahoma, Norman, OK 73019, USA; 3 Department of Radiology, University of Oklahoma Health Sciences Center, Oklahoma City, OK 73104, USA; theresa-thai@ouhsc.edu; 4 Department of Obstetrics and Gynecology, University of Oklahoma Health Sciences Center, Oklahoma City, OK 73104, USA

**Keywords:** radiomics, ovarian cancer, 2D and 3D features, incomplete 3D features, chemotherapy response prediction, precision medicine

## Abstract

**Background and Objective:** 2D and 3D tumor features are widely used in a variety of medical image analysis tasks. However, for chemotherapy response prediction, the effectiveness between different kinds of 2D and 3D features are not comprehensively assessed, especially in ovarian-cancer-related applications. This investigation aims to accomplish such a comprehensive evaluation. **Methods:** For this purpose, CT images were collected retrospectively from 188 advanced-stage ovarian cancer patients. All the metastatic tumors that occurred in each patient were segmented and then processed by a set of six filters. Next, three categories of features, namely geometric, density, and texture features, were calculated from both the filtered results and the original segmented tumors, generating a total of 1403 and 1595 features for the 2D and 3D tumors, respectively. In addition to the conventional single-slice 2D and full-volume 3D tumor features, we also computed the incomplete-3D tumor features, which were achieved by sequentially adding one individual CT slice and calculating the corresponding features. Support vector machine (SVM)-based prediction models were developed and optimized for each feature set. Five-fold cross-validation was used to assess the performance of each individual model. **Results:** The results show that the 2D feature-based model achieved an AUC (area under the ROC curve (receiver operating characteristic)) of 0.84 ± 0.02. When adding more slices, the AUC first increased to reach the maximum and then gradually decreased to 0.86 ± 0.02. The maximum AUC was yielded when adding two adjacent slices, with a value of 0.91 ± 0.01. **Conclusions:** This initial result provides meaningful information for optimizing machine learning-based decision-making support tools in the future.

## 1. Introduction

Ovarian cancer is the most aggressive malignancy in gynecologic oncology [1]. Given the difficulty in the early stage diagnosis of ovarian carcinoma, most patients are diagnosed with ovarian cancer at advanced stages [2], which can lead to the formation of metastatic tumors on various organs. To control these metastatic tumors, chemotherapy is the only effective treatment after the primary cytoreduction [3]. However, due to the nature of metastasis heterogeneity, the patients’ response to treatment varies largely. Thus, one challenge in current clinical practice is to select the appropriate chemotherapy before administrating the treatment. To address this, many studies have been conducted to identify biomarkers from either histologic or/and imaging data that might be associated with patient response to chemotherapy [4,5,6,7]. Among different technologies, imaging approaches have the advantage of evaluating the organ/tissue noninvasively with adequate spatial resolution. Moreover, being a widely available imaging modality with relatively operating low cost, CT is one of the most popular diagnostic tools in therapy response assessment [8]. However, the current criteria requires both pre-therapy and 6–8 week follow-up examinations, and the diameter-based evaluation causes low association with the clinical outcome [9].

Meanwhile, radiomics is an emerging technology that extracts a large amount of features from tumors segmented from various medical images [10,11,12]. These radiomics features are able to quantify the complex textures, shapes, as well as other tumor characteristics that are highly associated with carcinogenesis [13]. Therefore, this information can be beneficial in determining treatment options and predicting the tumor’s response to therapy [14]. For this technique, identifying effective radiomics features is critically important: 2D features are easier and faster to calculate but provide less information, while the 3D features use the entire 3D tumor volume and intuitively contain more comprehensive information. To this end, many studies have been conducted to explore the effectiveness of 2D and 3D features in different applications. The prognostic performance of 2D and 3D radiomics features in CT images of non-small cell lung cancer (NSCLC) was investigated in [15], which demonstrated that 2D features exhibited better performance, although both feature types had strong prognostic capability. One study compared the 2D and 3D radiomics features in characterizing gastric cancer [16], which revealed that models constructed with 2D radiomics features had comparable performance with those constructed with 3D features. In another study, Xu et al. [17] evaluated the prediction performance of 2D and 3D radiomics features in a multi-organ, multi-modality cancer study, and the authors concluded that 3D radiomics features were more effective. Lee et al. [18] compared the 2D and 3D texture features for discriminating between gastric cancer and normal gastric mucosa on CT images, and they revealed that 3D texture features were more effective than 2D features. Despite their clinical usefulness, these studies only compare features from the single-slice-based 2D tumor and the full-volume 3D tumor. Very few studies have been focused on the effectiveness of incomplete 3D tumors features, which contain only a portion of the image slices.

In this investigation, we hypothesized that the inclusion of multiple tumor slices would lead to a substantial enhancement in the predictive accuracy of our model, but it may also introduce more uncertainties that may adversely impact model accuracy. To verify this hypothesis, we extracted 2D radiomics features from a single slice of the tumors and then generated incomplete 3D features by sequentially adding the adjacent CT slices. Based on each individual feature pool, we optimized the same kind of predictive model and evaluated their performance, respectively. The details are presented in the following sections.

## 2. Materials and Methods

### 2.1. Database

In our study, we utilized a dataset of CT images involving 188 ovarian cancer patients. The CT images in the dataset were retrospectively collected from the University of Oklahoma Health Science Center (OUHSC). Subjects for this study included the following criteria: (1) they had recurrent ovarian/peritoneal/tubal carcinoma of high-grade histology (e.g., serous, endometrioid, and undifferentiated); (2) they received systemic chemotherapy treatment after primary cytoreduction. For each patient in the dataset, there were up to 5 metastatic tumors occurred in different organs, which were marked on the pre-therapy CT images. All these images were acquired under a routing clinical protocol. CT examinations were captured utilizing either a GE Light Speed VCT 64 or a GE Discovery 600 16-row detector machine with a tube voltage of 120 kVp. The currents were adjusted from 100 to 600 mA, depending on patient body size. The clinical outcome was also collected for model assessment. In this study, 6-month progression-free survival (PFS) was utilized, which a common index for assessing the efficacy of new cancer chemotherapies in clinical trials [19]. Accordingly, among all the collected 188 patients, 130 patients are responders with a 6-month PFS of ‘Yes’, whereas 58 patients are non-responders with little response to the treatments. The detailed demographic characteristics of our patients cohort can be found in Table 1. The entire study protocol was approved by the Institutional Review Board in the University (IRB13649).

### 2.2. Radiomics Feature Extraction

In this study, the tumor features were obtained from 2D and 3D pre-therapy CT slices. Each segmented tumor is depicted on a number of CT slices. The central slice has the largest 2D area, which was identified by radiologists. Accordingly, we first extracted the quantitative features only from the central slice to generate a 2D feature pool. We sequentially added the adjacent slices above and below the central slice until the tumor disappears. After adding one adjacent slice, we will create the corresponding incomplete 3D tumor feature pool (Table 2). We continued the generation of the corresponding incomplete 3D feature pool until the tumor disappears.

Before the feature computation, we developed a computer-aided image analysis scheme to segment each individual tumor from the CT images. The tumor segmentation procedure started at the central slice where the tumor was marked by a radiologist according to RECIST criteria [20]. The method then sequentially segmented the tumors on the adjacent slices until the tumor disappeared. For each slice, the tumor was segmented by a hybrid algorithm, which consists of two core algorithms: a region growth approach with adaptive thresholds [21] and a dynamic contour-seeker method [22]. The performance of this algorithm was assessed and validated by a number of our previous studies [23,24,25,26]. Given that metastatic tumors occurred on a number of human organs with high heterogeneity, the automated segmentation may not be able to generate the tumor contour with satisfactory accuracy. Thus, these segmentation results were visually evaluated by experienced researchers and were manually corrected if needed. The entire scheme was developed as an ImageJ plugin [27], which was equipped with a user friendly graphical user interface (GUI) to visualize the 2D and 3D tumors.

**Figure 1:**
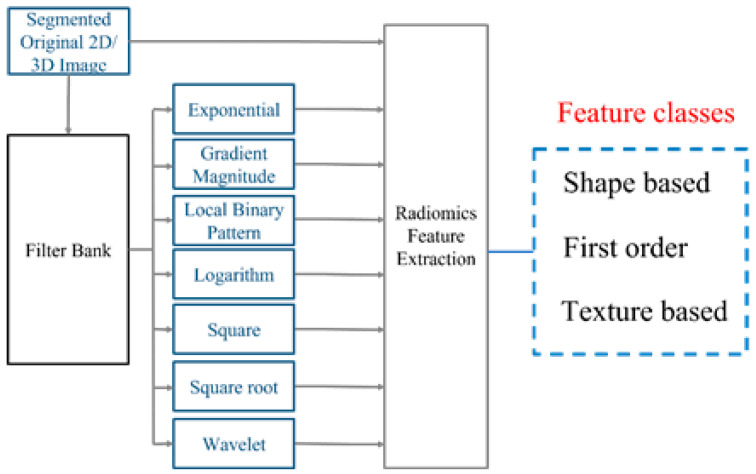
Overview of the feature extraction process [11].

Based on the segmented tumors, we generated the 2D and 3D feature pool using the following methods [11,12]. The feature computation scheme is based on pyradiomics [11], an open source platform that calculates features in accordance with the definitions in the imaging biomarker standardization initiative (IBSI) [12]. The general flowchart of the calculation is described in Figure 1. In this module, the segmented tumor was first processed by a number of operations including exponential, gradient magnitude, local binary pattern (LBP), logarithm (Log), square, square root, and wavelet (Coif1) filters. Next, the radiomics features were then calculated on the processed images as well as the original image. The features can be divided into 3 categories, i.e., geometric, density, and texture features (the details can be found in Supplementary Materials), which describe the tumor in various aspects [28]. Table 2 shows a breakdown of the number of features calculated for each image class, with the number of features per class ranging from 93 to 741. We calculated a total of 1595 and 1403 features for the 3D and 2D feature pools, respectively. Given that a total of up to five tumors were segmented by the scheme and the features were extracted on each individual tumor, the case-based value of each feature is generated by calculating the minimum $F_{min}=min\{F_{1},\;F_{2},\;F_{3},\;\ldots ,\;F_{N}\}$ of all the tumor-based values. The minimum value was selected on the basis of our empirical analysis, as it consistently demonstrates its effectiveness in providing a stable and conservative feature representation across the segmented tumors for each patient.

### 2.3. Develop Machine Learning-Based Models to Predict Tumor Response to Chemotherapy

Pearson correlation coefficient (PCC) and LASSO (Least Absolute Shrinkage and Selection Operator) approaches were used to reduce the dimensionality of our feature space. First, the PCC was calculated, and a heatmap was generated to investigate the feature dependency. After the computation, feature pairs with a Pearson correlation coefficient (PCC) exceeding the pre-set threshold were identified, and one of the correlated features was removed [29] to reduce the redundant information. Next, LASSO was applied on the rest of the feature pool to create an optimal feature cluster [30], as it has the capability to handle high-dimensional data with a limited number of observations [31]. Essentially, this method employs a variation of the least squares regression method that employs L1 regularization to produce sparse variable coefficients. Accordingly, LASSO constrains the sum of the absolute values of the regression weights to be smaller than a fixed threshold. Therefore, it improves model interpretability by removing variables irrelevant to the response variable and reduces the possibility of overfitting [32]. The LASSO objective function is defined in Equation (1).

(1) $$\sum_{i=1}^{n}{\left(y_{i}-\sum_{j}x_{ij}\beta_{j}\right)}^{2}+\lambda \;\sum_{j=1\;}^{p}\left|\beta_{j}\right|,\;\;\;\;\;\;\;\;\;subject\;to\;\sum_{j=1\;}^{p}\left|\beta_{j}\right|<t$$

In the above error function, t is the upper-bound threshold for the sum of the absolute values of feature weights β, and λ is a non-negative tuning parameter that regulates the degree of the penalty. We employed the LASSO-cross validation (LASSO-CV) approach to tune the LASSO parameters [33]. After applying LASSO, features with non-zero weights were chosen to be included in the model. Finally, we adopted the synthetic minority over-sampling technique (SMOTE) to add more non-responder samples and balance the dataset, which generates samples by interpolating between one selected minority sample with its nearest neighbors, which was performed using scikit-learn library [34].

Next, a support vector machine (SVM) was used to predict the tumor responses (i.e., 6-month PFS). SVM has been widely validated as an effective classifier in various medical imaging tasks [35,36,37,38]. The method constructs one or more hyperplanes to classify the feature vectors into two classes with minimal generalization error [39]. Mathematically, the SVM model is established by solving the following optimization problem: $\min\frac{1}{2}{∥w∥}^{2}+\frac{C}{2}{∥\xi ∥}_{2}^{2}$ with the constraint $y_{i}\left(w\Phi \left(x_{i}\right)+b_{i}\right)\geq 1-\xi_{i}$, $\xi_{i}\geq 0,\;\forall i$ or its dual form $\max W\left(\alpha \right)=e^{T}\alpha -\frac{1}{2}\alpha^{T}y_{i}y_{j}K\left(x_{i},x_{j}\right)\alpha$, $0\leq \;\alpha_{i}\leq C$, $y^{T}\alpha =0$. In the above formulae, $\left(x_{i},y_{j}\right)\;$is one pair of feature vector and output value, w and b are the normal vector and intercept of the discrimination hyperplane, $\xi_{i}$ is the slack variable, C is the adjusting coefficients for optimization errors, e is the unit vector, α is the Lagrange coefficient, and K represents kernel function $K\left(x_{i},x_{j}\right)=\exp\left(-\frac{∥x_{i}-x_{j}∥{}^{2}}{\sigma^{2}}\right)$ [40]. The model’s parameters (i.e., C and σ) will be tuned to achieve optimal values during the training process. The SVM parameter tuning was executed using grid search [41], which varies the parameters systematically to search through a predefined range of values and find the set that produced the most desirable performance metrics (e.g., accuracy). This process was conducted to ensure the robustness and reliability of our proposed approach, resulting in the selection of the most suitable parameters for the SVM model. Finally, each classifier was then trained and tested using a 5-fold cross-validation approach [42]. To evaluate the model performance, the receiver operating characteristic (ROC) curve was used [43], and the area under the ROC curve (AUC) was estimated for each model.

## 3. Results

**Figure 2:**
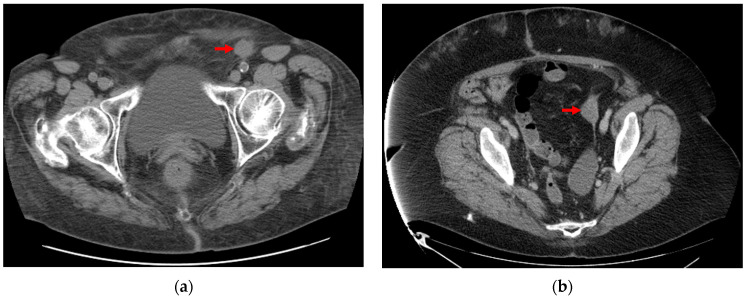
Sample tumor images from (**a**) responder case to treatment. (**b**) non-responder case.

**Figure 3:**
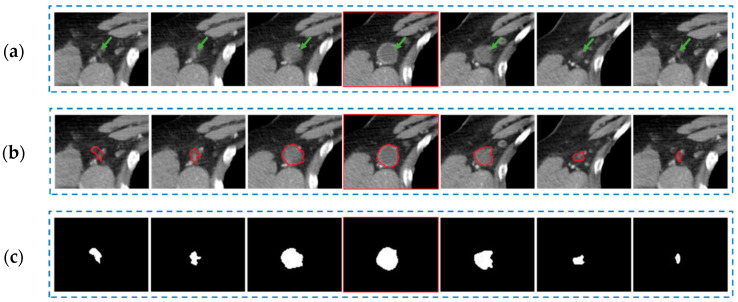
One example of the tumor segmentation process, in which the central slice is outlined with red color. (**a**) Identified tumor on the CT slices. (**b**) Tumors outlined using our developed CAD software. (**c**) Segmented tumor masks.

Figure 2a,b illustrates tumor images from a representative non-responder and a representative responder, respectively. Although there are no visible feature differences between the responder and non-responder, the computed radiomics feature can classify them into different groups. Figure 3 shows an example of metastatic tumor segmentation. The tumor has the largest area in the central slice, and its area gradually decreases until the tumor eventually disappears on the fourth neighboring slices of both sides (Figure 3a). Accordingly, the segmentation procedure starts with the central slice, where the tumor was identified by a radiologist or oncologist. Then, the segmentation continues on the adjacent slices until the tumor disappears (Figure 3b,c).

**Figure 4:**
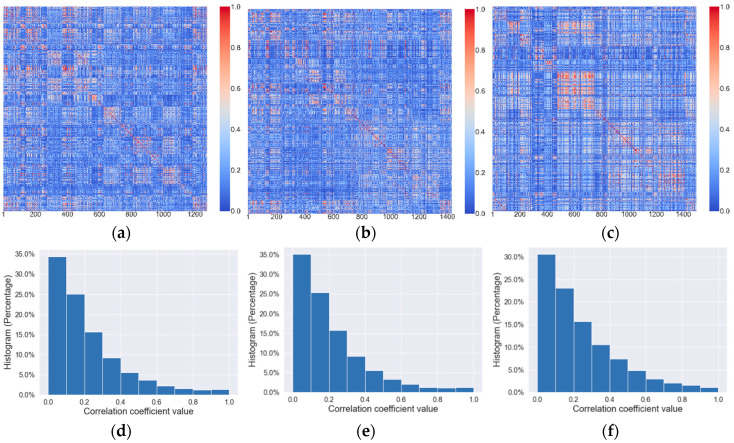
Feature heatmap of (**a**) 2D tumors, (**b**) incomplete 3D tumors, and (**c**) 3D tumor. The corresponding histograms were illustrated in (**d**–**f**) for 2D, incomplete 3D, and 3D features, respectively.

Figure 4a–c represents the heatmap of all the features extracted from 2D, incomplete 3D, and 3D tumor, respectively. The maps were produced by a total of 188 observations. Each entry in the map indicates the colorized Pearson correlation coefficient ranging from 0 (Blue) to 1 (red). The maps illustrate that most of the features extracted from 2D tumor masks have relatively low correlation. The similar pattern can be observed in the features obtained from incomplete 3D tumor masks. However, the correlation structure of the features extracted from whole 3D tumor masks was notably different, particularly in logarithm-based, exponential, square, and square root-based features, exhibiting higher correlation among different classes. The high correlation may be attributed to the enhancement of certain 3D structures when applying these filters, which leads to consistent value changes on some categories of the features. Furthermore, Figure 4d–f demonstrates the histograms of all feature categories for 2D, incomplete 3D, and 3D features. According to the histograms, more than 80% of correlation coefficient values for 2D and incomplete 3D features are less than 0.5, whereas a higher percentage of 3D features have correlation coefficients greater than 0.5. These results imply that 2D and incomplete-3D tumor features cover comprehensive tumor characteristics with low information redundancy.

**Figure 5:**
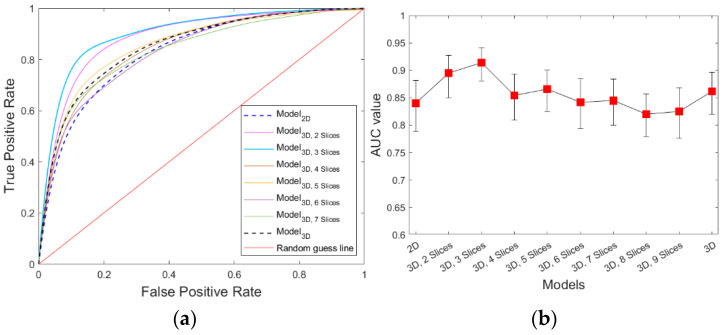
Performance comparison of the models for six-month PFS prediction (**a**) ROC curves of the prediction models. (**b**) The corresponding AUC values of ten prediction models (with 95% CI).

After applying the PCC and LASSO feature selection methods, we identified 115 two-dimensional features as the most effective feature cluster for further analysis. Similarly, 56–126 features were identified for different incomplete 3D tumor features pools, and 73 features were identified from the full 3D feature pool. As illustrated in Table 3, a minimum of 78% of the selected features can be categorized into texture-based features, while the remainder fall into the categories of shape or density features. When dividing the features based on the filters, we observe that the largest group of features is extracted from the wavelet transform-based filtered images, with the features calculated from LBP-filtered images forming the second most prominent group. Our models used these features as inputs to generate the prediction score. Figure 5 illustrates the ROC curve achieved by the models trained with one to nine number of slices of tumor mask and whole slices, while Table 4 summarizes the corresponding values of AUC and overall accuracy values. The results demonstrate that adding more slices to the central slice can increase the prediction performance, but only for a certain range of additional slices. The highest performance was achieved when the model was constructed with one central slice and two adjacent slices, with the model yielding an AUC of 0.91 ± 0.01 (95% confidence interval [0.88, 0.94]) and an overall predicting accuracy of 0.84 ± 0.03 (95% confidence interval [0.78, 0.88]). The 3D model with three and 5 adjacent slices were the second and third best performing models, which achieved an AUC values of 0.89 ± 0.01 and 0.86 ± 0.02, respectively. Model_3D, with an AUC of 0.86 ± 0.02 (95% confidence interval [0.82, 0.89]), performed better than Model_2D, with AUC of 0.84 ± 0.02 (95% confidence interval [0.78, 0.88]). All these trends are also illustrated in Figure 5b.

## 4. Discussion

In this study, we conducted a comprehensive evaluation of various radiomic feature extraction methods for predicting early response of ovarian cancer to chemotherapy. Our investigation focused on 2D, incomplete 3D, and 3D radiomic features and their ability to represent and discriminate the underlying tumor characteristics. Our findings demonstrate that the incomplete 3D features extracted from the central tumor slice and its two adjacent slices yielded better prediction accuracy and might be recommended as the preferred approach.

As compared to the previous studies, the most unique characteristic of our investigation is that we explored the performance of the features computed from incomplete 3D tumors, which contain a portion of the tumor slices. The comparison between the 2D or 3D features has been investigated by many research groups. In general, the 2D features are calculated from a single slice containing the maximal projection within the entire tumor. Accordingly, tumor segmentation and annotation are relatively easier, but some useful information is lost. For instance, it is difficult to accurately extract tumor volume, surface area, spherical disproportion, and other features that reflect geometric irregularities from a 2D tumor. On the other end, despite the comprehensive information, the 3D feature suffers from more information uncertainties. Given that it is very difficult to clearly define the boundary between the tumor region and its surround healthy tissues, there is no segmentation algorithm which is able to perfectly extract the tumor contour [44]. Moreover, the tumor heterogeneity may only be inside several central slices. The marginal slices may only contain suspicious surrounding tissue, and they may not have any information associated with the needed treatment responses. As a result, more uncertainties will be added on the segmented 3D tumors, which may adversely affect the performance of prediction models. Therefore, there is a trade-off between adding more useful information and uncertainties when sequentially increasing the adjacent slices. In this study, we first demonstrated that using the central and its two neighboring slices may achieve better performance than other situations. To the best of our knowledge, no similar studies have been performed on this topic before.

Moreover, our study highlights the importance of texture-based features and wavelet filtering for predicting treatment response. We observe that texture-based features were more informative, especially when they are computed from the wavelet transform-processed images. This could be attributed by the fact that texture-based features are able to capture the heterogeneity and complexity of the tumor microenvironment, which can provide insights into tumor biology and response to treatment. Wavelet transform is able to initially concentrate the tumor edge and texture information, while the other irrelevant information is discarded. Meanwhile, our study suggests that the number of slices used for CT image analysis can have an impact on the correlation structure of extracted radiomics features. The high correlation only occurs on the complete 3D tumor features from the tumor images processed by certain kinds of filters (e.g., logarithm, exponential, and square), which may be explained by the fact that such filters can enhance certain 3D structures, and these structures could lead to consistent value changes on some categories of the features.

Despite the promising results of our study, this study has the following limitations. First, the dataset used in our study only consisted of 188 patients, which were collected from a single institute. This single institute dataset may not be able to comprehensively represent the diversified population within the entire country, therefore the robustness of the results in this investigation should be further verified by a larger multi-institute patient cohort. Second, although the segmentation scheme was validated in our previous studies, the tumor segmentation accuracy was not considered. The segmentation error may also potentially introduce noise into our 3D features and consequently reduce the predictive performance [45]. Third, only radiomics features were used in the feature performance comparison. Other types of features, such as deep learning features, were not investigated. It would be worthwhile to explore the combination of deep learning features and radiomics features in future studies. In conclusion, our study may provide valuable information for the development of radiomics-based prediction models in the future.

## Figures and Tables

**Table 1:** Information of the patients in our dataset.

Patients’ Characteristics	6-Month PFS		*p*-Value
	Responders	Non-Responders	
Number of patients	130	58	
Age	63 ± 10	61 ± 10	0.24
Number of tumors	272	133	
Average tumor diameter (mm)	31.3 ± 19.3	31.5 ± 16.4	0.94

**Table 2:** Number of features calculated from original image and other processed versions of the image.

Image Type	2D	3D
Original	104	110
Exponential	93	93
Gradient Magnitude	93	93
Local binary pattern	93	279
Logarithm	93	93
Square	93	93
Square root	93	93
Wavelet	741	741
Total	1403	1595

**Table 3:** Summary of selected features by radiomics class type and filtering method.

Features	Radiomics Category			Filter			Total
	Shape	Density	Texture	Wavelet	LBP	Other	
2D	0	23	92	71	21	23	115
3D_2 Slices_	4	18	104	77	19	30	126
3D_3 Slices_	0	16	75	51	9	31	91
3D_4 Slices_	1	20	75	52	13	31	96
3D_5 Slices_	1	17	64	44	12	26	82
3D_6 Slices_	1	16	72	51	14	24	89
3D_7 Slices_	1	15	61	37	16	24	77
3D_8 Slices_	0	11	51	27	7	28	62
3D_9 Slices_	1	8	47	30	4	22	56
3D	1	8	64	34	13	26	73

**Table 4:** Performance comparison of the prediction models.

Model	AUC ± STD 95% CI	ACC ± STD 95% CI
2D	0.84 ± 0.02 [0.78, 0.88]	0.75 ± 0.03 [0.69, 0.80]
3D_2 Slices_	0.89 ± 0.01 [0.85, 0.92]	0.83 ± 0.02 [0.77, 0.87]
3D_3 Slices_	0.91 ± 0.01 [0.88, 0.94]	0.84 ± 0.02 [0.78, 0.88]
3D_4 Slices_	0.85 ± 0.02 [0.80, 0.89]	0.76 ± 0.02 [0.71, 0.82]
3D_5 Slices_	0.86 ± 0.02 [0.82, 0.90]	0.78 ± 0.02 [0.72, 0.83]
3D_6 Slices_	0.84 ± 0.02 [0.79, 0.88]	0.74 ± 0.03 [0.69, 0.79]
3D_7 Slices_	0.86 ± 0.02 [0.80, 0.88]	0.76 ± 0.03 [0.70, 0.80]
3D_8 Slices_	0.84 ± 0.02 [0.78, 0.86]	0.75 ± 0.03 [0.70, 0.80]
3D_9 Slices_	0.83 ± 0.01 [0.78, 0.87]	0.73 ± 0.02 [0.69, 0.78]
3D	0.86 ± 0.02 [0.82, 0.89]	0.77 ± 0.02 [0.72, 0.83]

## Data Availability

The dataset in this study is not publicly available.

## Supplementary Materials

The following supporting information can be downloaded at: https://www.mdpi.com/article/10.3390/bioengineering10111334/s1.
