# Supplementary material for: Evaluating the Effectiveness of 2D and 3D CT Image Features for Predicting Tumor Response to Chemotherapy
Source: Bioengineering (Basel). 2023 Nov 20;10(11):1334. doi: 10.3390/bioengineering10111334 (PMC10669238; doi:10.3390/bioengineering10111334)
Supplement: Supplementary file 1 [file bioengineering-10-01334-s001.zip › bioengineering-2659858-supplementary.pdf]

Supplementary Material

In this investigation, PyRadiomics tool was used to generate 2D and 3D radiomics features, which can be classified into three different groups, namely, shape-based, density-based, and texture-based feature. Table S1 shows a summary of all the used features, and the names of the features in each category are then listed. More detailed information regarding the feature definition can be found on the website: https://pyradiomics.readthedocs.io.

Table S1. Feature classes.

**Feature category**	**Feature sub-class**	**2D**	**3D**
Shape		10	16
Density		19	19
Texture	Gray Level Co-occurrence Matrix (GLCM)	24	24
	Gray Level Run Length Matrix (GLRLM)	16	16
	Gray Level Size Zone Matrix (GLSZM)	16	16
	Neighboring Gray Tone Difference Matrix (NGTDM)	5	5
	Gray Level Dependence Matrix (GLDM)	14	14
**Total**		**104**	**110**

Density features:

Energy, Total Energy, Entropy, Minimum, 10th percentile, 90th percentile, Maximum, Mean, Median, Interquartile Range, Range, Mean Absolute Deviation (MAD), Robust Mean Absolute Deviation (rMAD), Root Mean Squared (RMS), Standard Deviation, Variance, Skewness, Kurtosis, Uniformity

Shape features(2D):

Mesh Surface, Pixel Surface, Perimeter, Perimeter to Surface ratio, Sphericity, Spherical Disproportion, Maximum 2D diameter, Major Axis Length, Minor Axis Length, Elongation

3D Shape-based Features:

Mesh Volume, Voxel Volume, Surface Area, Surface Area to Volume ratio, Sphericity, Compactness1, Spherical Disproportion, Maximum 3D diameter, Maximum 2D diameter (Slice), Maximum 2D diameter (Column), Maximum 2D diameter (Row), Major Axis Length, Minor Axis Length, Least Axis Length, Elongation, Flatness

Gray Level Co-occurrence Matrix (GLCM) features:

Autocorrelation, Joint Average, Cluster Prominence, Cluster Shade, Cluster Tendency, Contrast, Correlation, Difference Average, Difference Entropy, Difference Variance, Joint Energy, Joint Entropy, Informational Measure of Correlation (IMC) 1, Informational Measure of Correlation (IMC) 2, Inverse Difference Moment (IDM), Maximal Correlation Coefficient (MCC), Inverse Difference Moment Normalized (IDMN), Inverse Difference (ID), Inverse Difference Normalized (IDN), Inverse Variance, Maximum Probability, Sum Average, Sum Entropy, Sum of Squares

Gray Level Run Length Matrix (GLRLM) Features:

Short Run Emphasis (SRE), Long Run Emphasis (LRE), Gray Level Non-Uniformity (GLN), Gray Level Non-Uniformity Normalized (GLNN), Run Length Non-Uniformity (RLN), Run Length Non-Uniformity Normalized (RLNN), Run Percentage (RP), Gray Level Variance (GLV), Run Variance (RV), Run Entropy (RE), Low Gray Level Run Emphasis (LGLRE), High Gray Level Run Emphasis (HGLRE),

Short Run Low Gray Level Emphasis (SRLGLE), Short Run High Gray Level Emphasis (SRHGLE), Long Run Low Gray Level Emphasis (LRLGLE), Long Run High Gray Level Emphasis (LRHGLE)

Gray Level Size Zone Matrix (GLSZM) Features:

Small Area Emphasis (SAE), Large Area Emphasis (LAE), Gray Level Non-Uniformity (GLN), Gray Level Non-Uniformity Normalized (GLNN), Size-Zone Non-Uniformity (SZN), Size-Zone Non-Uniformity Normalized (SZNN), Zone Percentage (ZP), Gray Level Variance (GLV), Zone Variance (ZV), Zone Entropy (ZE), Low Gray Level Zone Emphasis (LGLZE), High Gray Level Zone Emphasis (HGLZE), Small Area Low Gray Level Emphasis (SALGLE), Small Area High Gray Level Emphasis (SAHGLE), Large Area Low Gray Level Emphasis (LALGLE), Large Area High Gray Level Emphasis (LAHGLE)

Neighboring Gray Tone Difference Matrix (NGTDM) Features:

Coarseness, Contrast, Busyness, Complexity, Strength

Gray Level Dependence Matrix (GLDM) Features:

Small Dependence Emphasis (SDE), Large Dependence Emphasis (LDE). Gray Level Non-Uniformity (GLN), Dependence Non-Uniformity (DN), Dependence Non-Uniformity Normalized (DNN), Gray Level Variance (GLV), Dependence Variance (DV), Dependence Entropy (DE), Low Gray Level Emphasis (LGLE), High Gray Level Emphasis (HGLE), Small Dependence Low Gray Level Emphasis (SDLGLE), Small Dependence High Gray Level Emphasis (SDHGLE), Large Dependence Low Gray Level Emphasis (LDLGLE), Large Dependence High Gray Level Emphasis (LDHGLE)
